# Development of Parasitic Organs of a Stem Holoparasitic Plant in Genus *Cuscuta*

**DOI:** 10.3389/fpls.2019.01435

**Published:** 2019-11-12

**Authors:** Kohki Shimizu, Koh Aoki

**Affiliations:** Graduate School of Life and Environmental Sciences, Osaka Prefecture University, Sakai, Japan

**Keywords:** attachment cells, conductive cells, *Cuscuta*, haustorium, host factors, intrusive cells, parasitic organs, parasitic plants

## Abstract

Parasitic plants infect a broad range of plant species including economically important crops. They survive by absorbing water, minerals, and photosynthates from their hosts. To support their way of life, parasitic plants generally establish parasitic organs that allow them to attach to their hosts and to efficiently absorb substances from the vascular system of the host. Here, we summarize the recent progress in understanding the mechanisms underlying the formation of these parasitic organs, focusing on the process depicted in the stem holoparasitic genus, *Cuscuta*. An attachment structure called “holdfast” on the stem surface is induced by the light and contact stimuli. Concomitantly with holdfast formation, development of an intrusive structure called haustorium initiates in the inner cortex of the *Cuscuta* stem, and it elongates through apoplastic space of the host tissue. When haustoria reaches to host vascular tissues, they begin to form vascular conductive elements to connect vascular tissue of *Cuscuta* stem to those of host. Recent studies have shown parasite-host interaction in the interfacial cell wall, and regulation of development of these parasitic structures in molecular level. We also briefly summarize the role of host receptor in the control of compatibility between *Cuscuta* and hosts, on which occurrence of attachment structure depends, and the role of plant-to-plant transfer of long-distance signals after the establishment of conductive structure.

## Introduction

A group of plants called “parasitic plants” have been reported to consist of 4000 or more species, which is equivalent to approximately 1% of flowering plants, and are found all over the world (Nickrent, 2002). In many cases, the host range of a parasitic plant is wide, infesting many plant species including economically important crops (Lanini and Kogan, 2005). Thus, parasitic plants cause serious damage to crop production.

Parasitic plants can be classified into two classes: hemiparasites that retain the ability to perform photosynthesis, and holoparasites that have little or no photosynthetic capability. Consequently, holoparasites need to live a heterotrophic lifestyle by depriving nutrients and water from host plants Heide-Jørgensen, 2008). Parasitic plants belonging to the genus *Cuscuta*, a member of the family Convolvulaceae, infest a broad range of hosts and have been used as a model for the study of stem parasitic plants. The genus *Cuscuta* has been reported to consist of more than 150 species (Yuncker, 1932), and belong to the holoparasitic class with degenerated leaves and roots, and, as they do not perform photosynthesis, depend entirely on host plants for nutrients and water. To understand *Cuscuta* at genetic level and to prevent damage to crop production, the whole genomes of *Cuscuta australis* (Sun et al., 2018) and *C. campestris* (Vogel et al., 2018) have been recently sequenced.

After germination, *Cuscuta* extends a thread-like shoot. During shoot extension, the extending stem performs a swinging movement to increase the probability of contact with the host plant (Tada et al., 1996). It has been reported that *Cuscuta* perceives volatiles emitted from the host and extends toward it (Runyon et al., 2006). If *Cuscuta* cannot find a host plant, it will die in about 2 weeks after germination.

**Figure 1:**
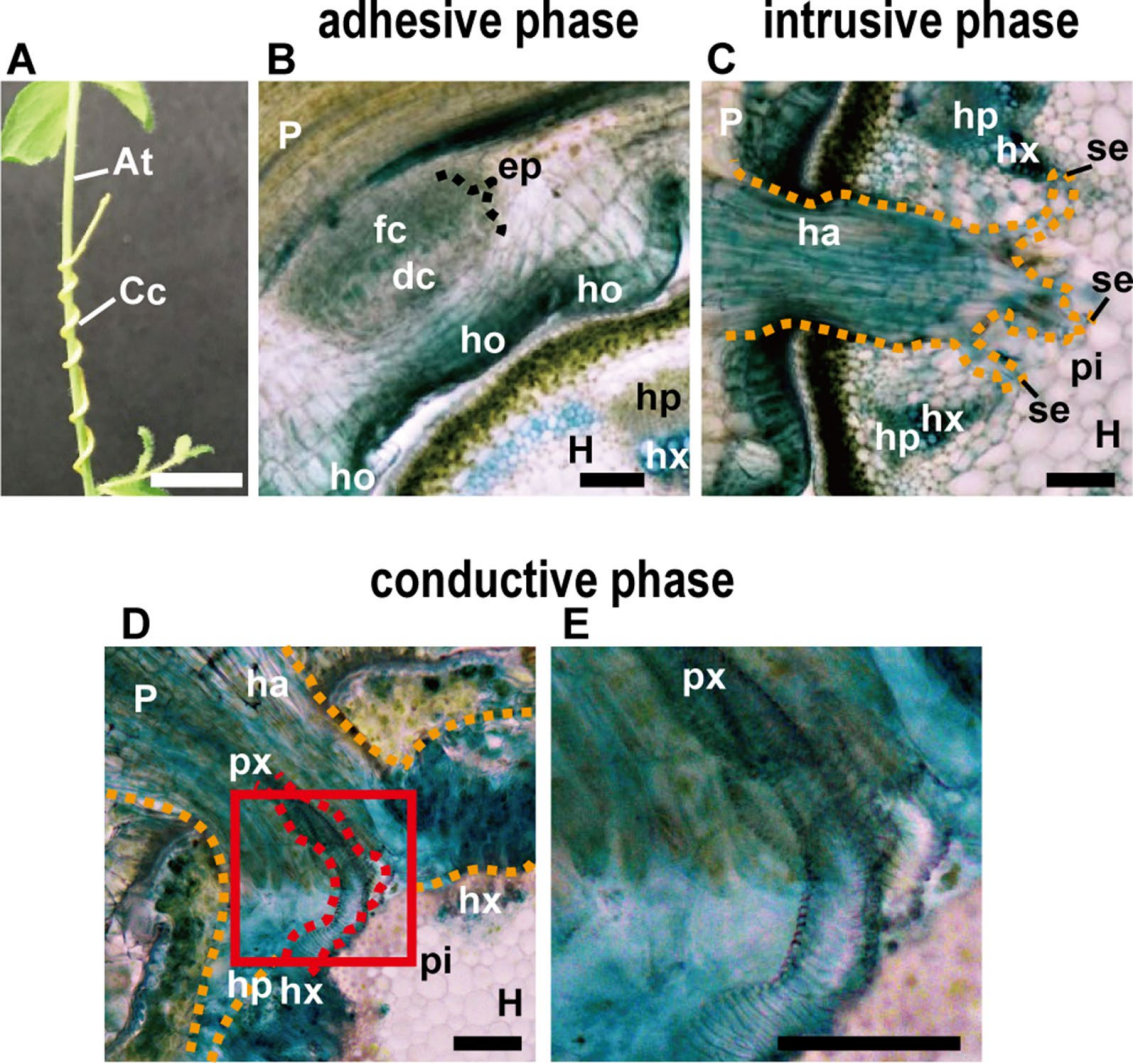
**(A)** Appearance of parasitic site formed between *Cuscuta campestris* (Cc) and *Arabidopsis thaliana* (At) from the outside. *C. campestris* coils around the inflorescence stem of Arabidopsis. Scale bar, 1 cm. **(B–E)** Transverse sections of the three phases of parasitic processes of *Cuscuta*. Scale bars, 200 μm. **(B)** Adhesive phase. Holdfast (ho) is formed on the host-attaching surface of *C. campestris*. Prehaustorium develops in the inner cortex of the stem right behind holdfast. In the endophyte primordium (ep), digitate cells (dc) and file cells (fc) differentiate and start to elongate. **(C)** Intrusive phase. Haustorium (ha) intrudes in the cortex of the host stem. It sometimes reaches to the pith (pi). **(D)** Conductive phase. **(E)** Area in the red square in **(D)** is magnified. Vascular conductive elements (px) are formed in the haustorim. P, parasite; H, host; ha, haustorium; hp, host phloem; hx, host xylem; px, parasite xylem; pi, pith; se, searching hypha; orange dotted line, outline of haustorium; red dotted line, outline of parasite xylem. In all panels, 200-μm-thick micro-slicer sections were stained with toluidine blue.

After contact with the host, the stem of *Cuscuta* forms a counterclockwise coil around the stem of the host (**Figure 1A**). The coiling behavior has been shown to be induced by the cooperative effects of far-red/blue light and tactile stimuli (Lane and Kasperbauer, 1965; Tada et al., 1996; Furuhashi et al., 1997). Effect of far-red light on the coiling of *C. japonica* was canceled by red light, suggesting the involvement of phytochrome (Furuhashi et al., 1997). Coiling and projection of haustoria of *C. japonica* can be induced by placing the stem between two glass plates to apply contact pressure under far-red or blue light, but was not induced under red- or white light, suggesting the cooperative effect of light and tactile stimuli (Tada et al., 1996).

After coiling on the host stem, a series of organogenesis occurs to establish a parasitic connection, including formation of an adhesive disc-like organ, referred to as a “holdfast” on the surface of the *Cuscuta* stem in contact with the host stem, and the development of a “haustorium” that intrudes into the host stem and finally makes vascular connection to the xylem vessels and phloem sieve tubes of the host (Yoshida et al., 2016). In this review, we describe the mechanisms underlying the formation of these parasitic organs, and propose hypotheses for the involvement of putative host factors. Comparison of *Cuscuta* with other well-studied root parasites belonging to Orobanchaceae that are taxonomically distant from *Cuscuta* highlight diversity with respect to the structure and function of the parasitic organs. We also briefly summarize the role of host receptor in the control of compatibility between *Cuscuta* and hosts, and the role of plant-to-plant transfer of long-distance signals after the establishment of conductive structure.

## Organogenesis Associated With Parasitic Connection

The parasitic processes of *Cuscuta* can be classified into three phases; the adhesive, intrusive, and conductive phases (**Figures 1A–E**) (Heide-Jørgensen, 2008). In the adhesive phase, a specialized adhesive organ called the holdfast is formed in the *Cuscuta* stem in contact with the stem of the host plant. Holdfast is formed essentially by the elongation of cells in the epidermal and cortical layers of *Cuscuta* stem, and characterized by the presence of secretory cells that secrete adhesive compounds (Heide-Jørgensen, 2008). In the intrusive phase, *Cuscuta* develops a specialized intrusive organ called the haustorium. When the haustorium reaches the vascular tissues of the host, a specific group of haustorial cells differentiate into vascular conductive cells and *Cuscuta* proceeds into the conductive phase. In the conductive phase, *Cuscuta* exchanges various information molecules with the host, as well as absorbs water and nutrients.

### Adhesive Phase

**Figure 2:**
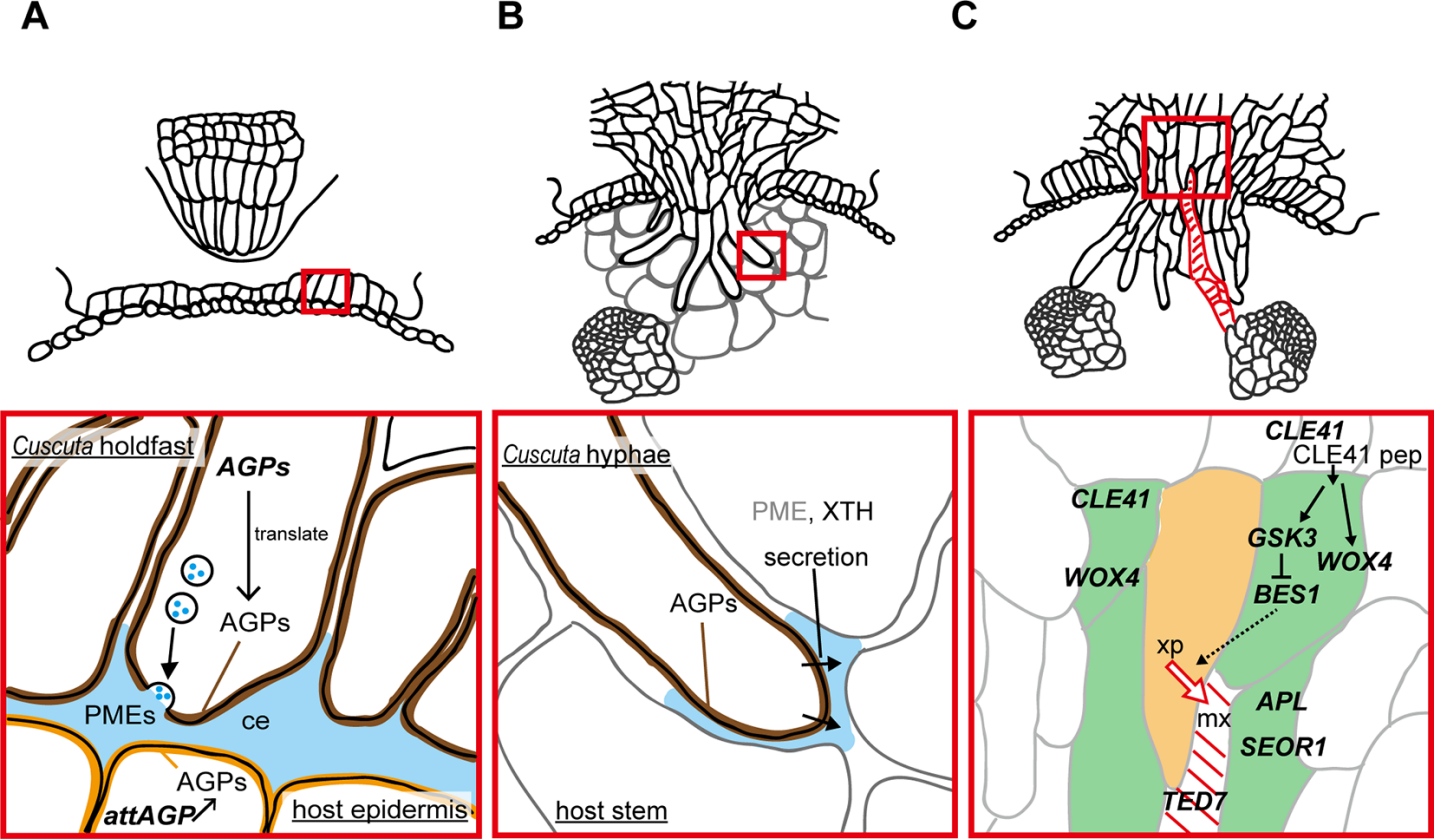
Functions of enzymes and genes associated with the parasitic processes. Panels in the bottom show magnified views of the areas in red squares in panels on the top. **(A)** Putative function of cell wall-modifying enzymes secreted from holdfast in the adhesive phase. Holdfast cells tighten the adhesion by pectin-rich cement (ce, blue). It has been shown that holdfast cells of *Cuscuta campestris* contain numerous secretion vesicles containing the components of cell-wall-loosening complexes. Pectin methylesterases (PMEs) are probably secreted to tighten the adhesion of *Cuscuta* to host. Specific members of genes encoding arabinogalactan proteins (*AGPs*) are expressed in searching hyphae, and accumulate AGP proteins (brown). AGP also have roles in host cell surface (orange) in the adhesion of parasite (Albert et al., 2006). **(B)** Secretion of cell wall-modifying enzymes to the cell walls adjacent to searching hyphae in the intrusive phase. Xyloglucan endotransglucosylation (XET) activity of XTH was detected in interface (blue) at the tip of haustoria of *C. reflexa* (Olsen and Krause, 2017). In *C. campestris*, searching hyphae-specific expression of *FASCICLIN-LIKE* genes causes the accumulation of AGPs in the interfacial cell walls surrounding searching hypha cells (brown) (Hozumi et al., 2017) Exact role of AGPs in the intrusive phase is still unknown. **(C)** Expression of genes associated with differentiation of vascular elements during the transition from intrusive phase to conductive phase in haustorim of *Cuscuta japonica*. Green, procambium/phloem region, orange, xylem precursor (xp), red diagonal lines, mature xylem vessel (mx). *WOX4*, *WUSCHEL RELATED HOMEOBOX 4*; *CLE41*, *CLAVATA3/EMBRYO SURROUNDING REGION-RELATED 41*; *GSK3*, GLYCOGEN SYNTHASE KINASE 3; *BES1*, *BRI1-EMS-SUPPRESSOR 1; TED7*, *TRACHEARY ELEMENT DIFFERENTIATION-RELATED 7*; *APL*, *ALTERED PHLOEM DEVELOPMENT*; *SEOR1*, *SIEVE ELEMENT OCCLUSION-RELATED 1*.

After coiling (**Figure 1A**), epidermal cells of the *Cuscuta* stem in contact with the host elongate toward the contacting surface of the host epidermis and divide anticlinally to become digitate in form (**Figure 1B**; Vaughn, 2002). Tight adhesion between *Cuscuta* and the host can be achieved by secretion of adhesive substances and elongation of cells toward the host surface. The divided epidermal cells of *Cuscuta campestris* (synonymous with *Cuscuta pentagona*, Costea et al., 2015) secrete pectin-rich adhesive (cement) to make a tight adhesion (Vaughn, 2002). Homogalacturonan, which constitutes up to 65% of cell wall pectin, is synthesized in a methyl-esterified form (Ridley et al., 2001). Methyl esters are removed enzymatically by pectin methylesterases (PMEs) from homogalacturonan (Micheli, 2001; Pelloux et al., 2007). Several studies using Arabidopsis have shown that low-esterified pectin is responsible for the organ adhesion (Sieber et al., 2000; Sala et al., 2019). In the epidermal layer of *Cuscuta* holdfast, immunolabeling of cell wall using antibodies against low-esterified homogalacturonan, such as JIM5 and LM19, is relatively stronger than that using antibodies against high-esterified homogalacturonan, such as JIM7 and LM20 (Vaughn, 2002; Johnsen et al., 2015; Hozumi et al., 2017). These result suggested that low-esterified homogalacturonan is responsible for the adhesion of *Cuscuta* to the hosts (**Figure 2A**).

Arabinogalactan proteins (AGPs) have been reported to be found in common in many adhesion-based mechanisms (Bowling and Vaughn, 2008; Huang et al., 2016). Implication for the involvement of AGPs in *Cuscuta* adhesion to the host was obtained by accumulation of AGP in the surface of the holdfast (**Figure 2A**). Staining with LM2 antibody which recognizes carbohydrate moiety of AGPs demonstrate that AGPs accumulate in epidermal cells on the surface of holdfasts of *Cuscuta reflexa* (Striberny and Krause, 2015) and *C. campestris* (Hozumi et al., 2017). Staining with Yariv reagents and LM6 antibody further support AGPs accumulation in epidermal cells of holdfasts of *C. campestris* (Hozumi et al., 2017). Accumulation of AGPs are due to the cell type-specific expression of a subset of fasciclin-like family member genes, *CcFLA7*, *16* and *17*. Accumulation of AGP on the contacting surface was also reported for host plants (Albert et al., 2006; Striberny and Krause, 2015). Contact of *Cuscuta reflexa* to the surface of tomato stem induces the expression of *attAGP* in tomato (Albert et al., 2006). Expression levels of tomato *attAGP* was positively correlated with the force of attachment. This result suggests a positive contribution of AGPs to parasite-host attachment (Albert et al., 2006). However, exact role of AGP in parasite-host attachment is still unknown.

To contact tightly to the host surface, divided epidermal cells of holdfast elongate toward the host surface (**Figures 1B** and **2A**). Outgrowth of the epidermal cells of the holdfast contributes to tightening of the adhesion by accommodating the surface of the host plant (Vaughn, 2002). The surface of the holdfast, which was in a pointed fingerlike extension form, becomes flat or rounded (**Figure 2A**). This malleability of the holdfast epidermis facilitates the formation of tight seal with the host surface (Vaughn, 2002). Identity of the elongating cell was referred to as a secretary trichome which contains a large number of secretary vesicles (Vaughn, 2002). Epidermal cells of the *Cuscuta* holdfast likely to share common developmental mechanisms with root hair (Ishida et al., 2008) or leaf trichome (Wang et al., 2019), although the expression of marker genes for these types of cells have not been demonstrated yet.

### Initiation of Intrusive Phase

The intrusive phase is characterized by the development of a haustorium (**Figure 1C**). To be accurate, primordia of haustoria have already been initiated in the adhesive phase. When *Cuscuta* develops holdfasts after contact to the host’s stem, the precursor of mature haustorium, or so-called prehaustorium, is differentiated in the cortex near the vascular cylinders right behind the holdfast (**Figure 1B**).

Initiation of the haustorium development appears to be a host-independent process. Development of haustoria in *Cuscuta* species can be induced even when *Cuscuta* coiled to non-biological object (Tada et al., 1996; Heide-Jørgensen, 2008; Hong et al., 2011). Microscopic studies have shown that meristem cells of haustorium develop simultaneously with the development of holdfast (Lee and Lee, 1989; Lee, 2007; Heide-Jørgensen, 2008). Initiation of haustorium development requires far-red light, and also blue light even though the effect is weaker than far-red light (Furuhashi et al., 1995), and by contact stimuli concomitantly applied with light (Tada et al., 1996; Furuhashi et al., 1997). Red or white light did not induce haustorium, and haustorium induction by far-red light can be cancelled by the following red light, suggesting the involvement of phytochrome in the regulation of haustorium development (Tada et al., 1996; Furuhashi et al., 1997). Cryptochrome is involved in blue light perception (Cashmore et al., 1999), and mechanosensitive ion channels are likely to be involved in the perception of contact stimuli (Hamilton et al., 2015; however, primary receptors for these stimuli have not been identified yet in *Cuscuta*.

Cytokinin has been reported to induce haustorium of *Cuscuta reflexa* in the absence of the host (Ramasubramanian et al., 1988), and in the dark (Haidar et al., 1998). These results imply that cytokinin may be a downstream signal of light and contact stimuli.

Genetic networks involved in the initiation of haustorium development have not yet been elucidated. Haustoria of *Cuscuta* develop as lateral protrusion of parasite stems, thus classified as “lateral haustoria” (Joel, 2013; Yoshida et al., 2016). Mechanisms involved in the formation of lateral organs, such as lateral roots and adventitious roots, have been studied in detail in Arabidopsis (Hu and Xu, 2016; Liu et al., 2018; Ibáñez et al., 2019; Lee et al., 2019), which may serve as a reference model for the initiation of *Cuscuta* haustorium.

### Development of Haustorium

**Figure 3:**
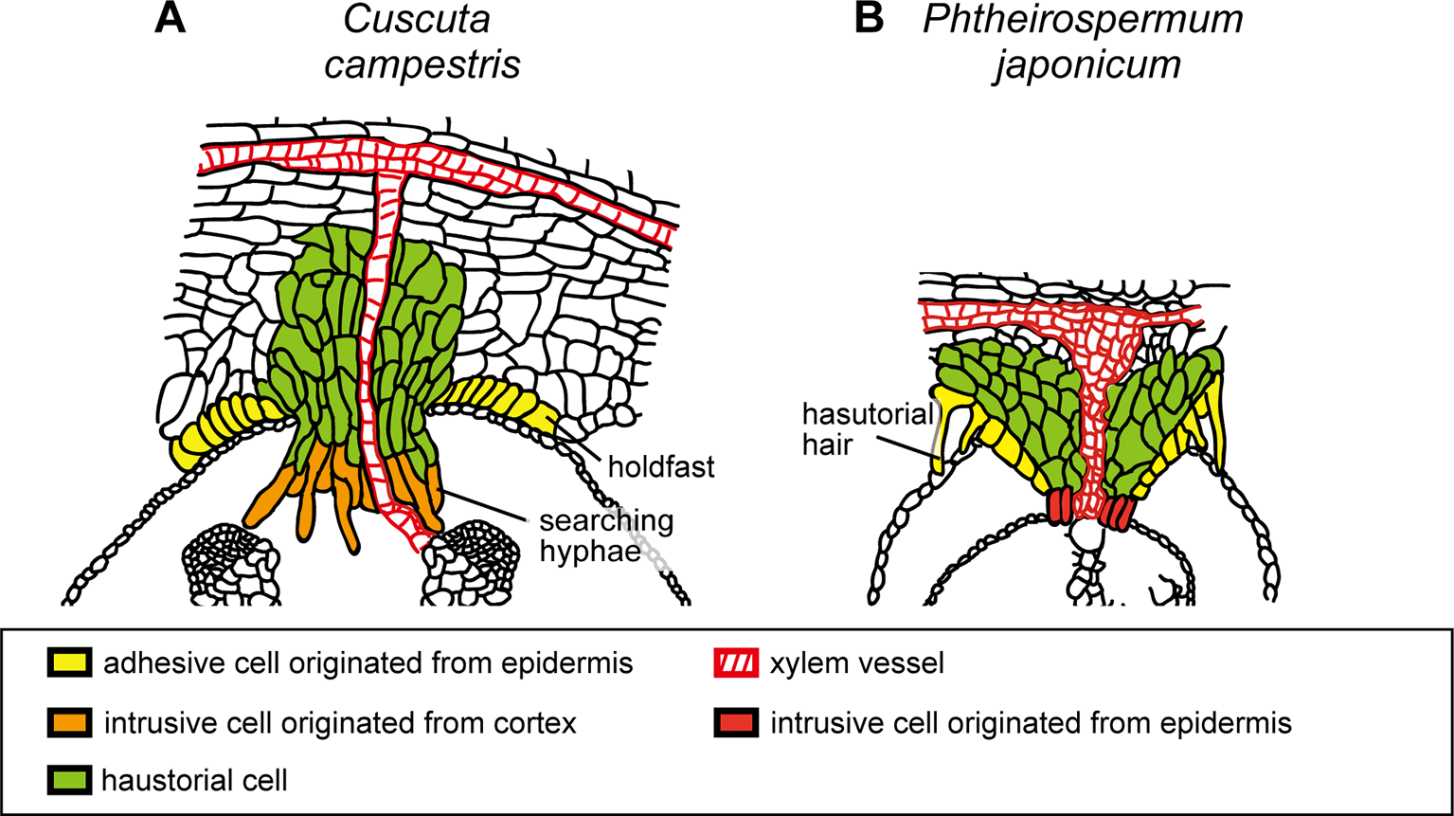
Schematic illustration of the structures of haustoria of *Cuscuta campestris* and *Phtheirospermum japonicum*. **(A)** *Cuscuta campestris*, a holoparasitic plant belonging to Convolvulaceae, develops lateral haustoria. **(B)** *Phtheirospermum japonicum*, a hemiparasitic plant belonging to Orobanchaceae, develops lateral haustoria. Holdfast of *C. campestris* and haustorial hair of *P. japonicum* are likely to be analogous that develop from epidermal cells and contribute to the adhesion of parasite to host. Intrusive cells of *C. campestris*, searching hyphae, develop from digitate cells which have been differentiated from the cortex or endodermal cells of the stem. On the other hand, intrusive cells of *P. japonicum* are shown to be differentiated from the epidermal cells.

Initial cells of *Cuscuta* haustorium are formed in the stem inner cortex, which then divide anticlinally and periclinally to give rise meristem cells (Lee and Lee, 1989; Lee, 2007). Meristem cells are then organized in “endophyte primordium” consisting of two cell types; elongate digitate cells and smaller file cells, before intruding into the host (**Figure 1B**; Lee, 2007). These cells become intrusive and force their ways through the stem cortex cells in front of them, the epidermal layer of its own stem and the epidermal layer and the cortex of the host. Cells in between the meristem and the stele also divide and form tabular cells, which are added to the file cell layer. This morphological observation suggests that the intrusive cells of *Cuscuta* originate from the cortex. This is different from the case of *Phtheirospermum japonicum*, a root hemiparasitic plant belonging to Orobancheceae, whose intrusive cells have been shown to originate from the root epidermal cells (Wakatake et al., 2018) (**Figures 3A, B**). During intrusive growth in the host’s cortex, intrusive cells advance in the apoplastic space by pushing the cells. At the front of haustorial intrusive part, the elongate digitate cells search for the host’s vascular tissues, and, thus are called “searching hyphae” (**Figure 1C**; Vaughn, 2003).

Intrusive cells grow through apoplastic space by pushing host cells aside, rather than by crushing them. *Cuscuta* secretes enzymes to the interfacial cell walls to loosen the cell wall and aid the elongation of intrusive cells in the apoplastic space. In *Cuscuta reflexa*, haustorium-specific expression of gene encoding a cysteine protease, namely cuscutain, were reported (Bleischwitz et al., 2010). In the parasitic interface of *C. japonica* and the host, *Glycine max*, expression of *C. japonica* genes encoding cell wall degrading- and modifying- enzymes, such as PME, pectate lyase, polygalacturonase, and xyloglucan endotransglucosylase/hydrolase (XTH) were up-regulated (Ikeue et al., 2015). In the far-red light-induced haustoria of *C. reflexa* and *C. gronovii*, two *XTH* genes have shown to be up-regulated (Olsen et al., 2016b). One of the two enzymatic activities of XTH, xyloglucan endotransglucosylation (XET), were secreted from haustoria, and localized at the host-parasite border of the endophytically growing haustoria of *C. reflexa*, *C. campestris* and *C. platyloba* (Olsen and Krause, 2017). Because XET activity of XTH grafts the reducing end of the cleaved xyloglucan onto an acceptor xyloglucan chain (Rose et al., 2002; Olsen et al., 2016a), these results indicate that *Cuscuta* XTHs play a role in invading growth of haustoria (**Figure 2B**).

In addition to cell wall modifying enzymes, searching hyphae of *C. campestris* and *C. japonica*, which develop on the haustorial tip, accumulate AGPs in the cell surface (**Figure 2B**). In *C. campestris*, hyphal AGP accumulation is accompanied by the expression of hyphae-specific *FASCICLIN-LIKE* family members (Hozumi et al., 2017). However, roles of hyphal AGP in intrusive growth is still unclear.

### Transition From Intrusive Phase to Conductive Phase

Once searching hyphae reach the host’s vascular tissues, the invasion process is almost complete. Searching hyphae acquire identities as xylem- and phloem-conductive elements (**Figures 1D, E**; Vaughn, 2006; Shimizu et al., 2018), which is concomitantly associated by the differentiation of vascular conducting elements in the center of haustorium (**Figure 2C**). Cells that have a procambium-attribute, from which vascular elements are differentiated, have emerged before contact with the host’s vascular elements. Cells with a procambium-attribute can be identified by the expression of *WUSCHEL RELATED HOMEOBOX 4* (*WOX4*) (Hirakawa et al., 2010). Expression of *C. japonica WOX4*, *CjWOX4*, was detected in the central region of the basal haustorium, and in cells surrounding the precursor cells which later differentiate into xylem vessels (**Figure 2C**; Shimizu et al., 2018).

Differentiation of searching hyphae into xylem starts near the tip. Searching hyphae penetrate into host xylem vessels through the pits, and starts a series of changes to differentiate xylem vessels (Vaughn, 2006). Xylem differentiation in haustoria of *C. japonica* include many processes in common with those elucidated in vascular tissues of model plants (Ito et al., 2006; Hirakawa et al., 2008). Before the onset of xylem differentiation, high expression of *C. japonica CLAVATA3/EMBRYO SURROUNDING REGION-RELATED 41* (*CjCLE41*), and CLE41 peptide is likely to be secreted to repress the differentiation of the procambium-like cells into tracheary elements. Expression of *CjCLE41* begins to decrease upon the onset of xylem differentiation, which probably down-regulates the kinase activity of GLYCOGEN SYNTHASE KINASE 3 (GSK3) protein. Down-regulation of GSK3 releases the expression of *BRI1-EMS-SUPPRESSOR 1* (*CjBES1*) from the deactivated state. Consequently, activated *CjBES1* expression induces the xylem differentiation processes (Shimizu et al., 2018). Expression of the gene specific to developing xylem vessels, *TRACHEARY ELEMENT DIFFERENTIATION-RELATED 7* (*CjTED7*), is under the detection limit before the onset of xylem differentiation, whereas up-regulated with xylem vessel formation (Shimizu et al., 2018).

Compared to xylem, differentiation of phloem in haustoria has been rather controversial and appears to differ from species to species. In *C. japonica*, marker genes of phloem companion cell, *ALTERED PHLOEM DEVELOPMENT* (*CjAPL*; Bonke et al., 2003), and of developing sieve elements, *SIEVE ELEMENT OCCLUSION-RELATED 1* (*CjSEOR1*; Knoblauch et al., 2014), were detected in the intruding haustoria (**Figure 2C**; Shimizu et al., 2018). Substances from the host’s sieve tube to *Cuscuta* translocate in distinct arrays of conductive cells (Birschwilks et al., 2006; Shimizu et al., 2018), indicating that phloem conductive cells develop in haustoria and are symplastically separated from surrounding cells. However, *in situ* hybridization for *CjCLE41*, whose Arabidopsis ortholog was expressed in phloem cells and adjacent pericycles (Hirakawa et al., 2008), demonstrated that it is expressed in cells overlapping with the region where *CjWOX4* is expressed (Shimizu et al., 2018). This incomplete compartmentalization implies immaturity of haustorial phloem relative to that in the conventional vascular bundles.

Differentiation processes of vascular cells in *Cuscuta* haustoria contain common and different processes compared to those in other parasitic plants. In *Phtheirospermum japonicum*, expression of procambium-specific genes, *PjWOX4*, *HOMEOBOX PROTEIN 8* (*PjHB8*) and *PjHB1*, were detected before the formation of xylem vessels, (Wakatake et al., 2018). This demonstrates that the development of procambium-like cells precedes the differentiation of haustorial vascular cells, as seen in *C. japonica*. On the other hand, organization of haustorial vascular cells appears to be different from that of *C. japonica*. Although the presence of xylem vessels are apparent, absence of *AtAPL* promoter activity, which is expressed in phloem, in haustoria suggest that phloem does not develops in *P. japonicum* haustoria (Spallek et al., 2017; Wakatake et al., 2018). Similarly, immaturity of the phloem is also shown in the haustoria of root holoparasitic plant, *Phelipanche aegyptiaca*, which also belongs to Orobanchaceae (Ekawa and Aoki, 2017). On the other hand, formation of mature sieve elements in haustoria has been reported for *Orobanche crenata* and *O. cumana* (Dörr and Kollmann, 1995; Krupp et al., 2019). These results, together with haustorial development of *Cuscuta* described in this section, suggest that development of procambium-like cells and haustorial xylem vessels are observed in common, on the other hand, development of phloem is different between different parasitic plants species. Mechanisms that bring diversity to phloem development have not been elucidated yet.

### Conductive Phase

**Figure 4:**
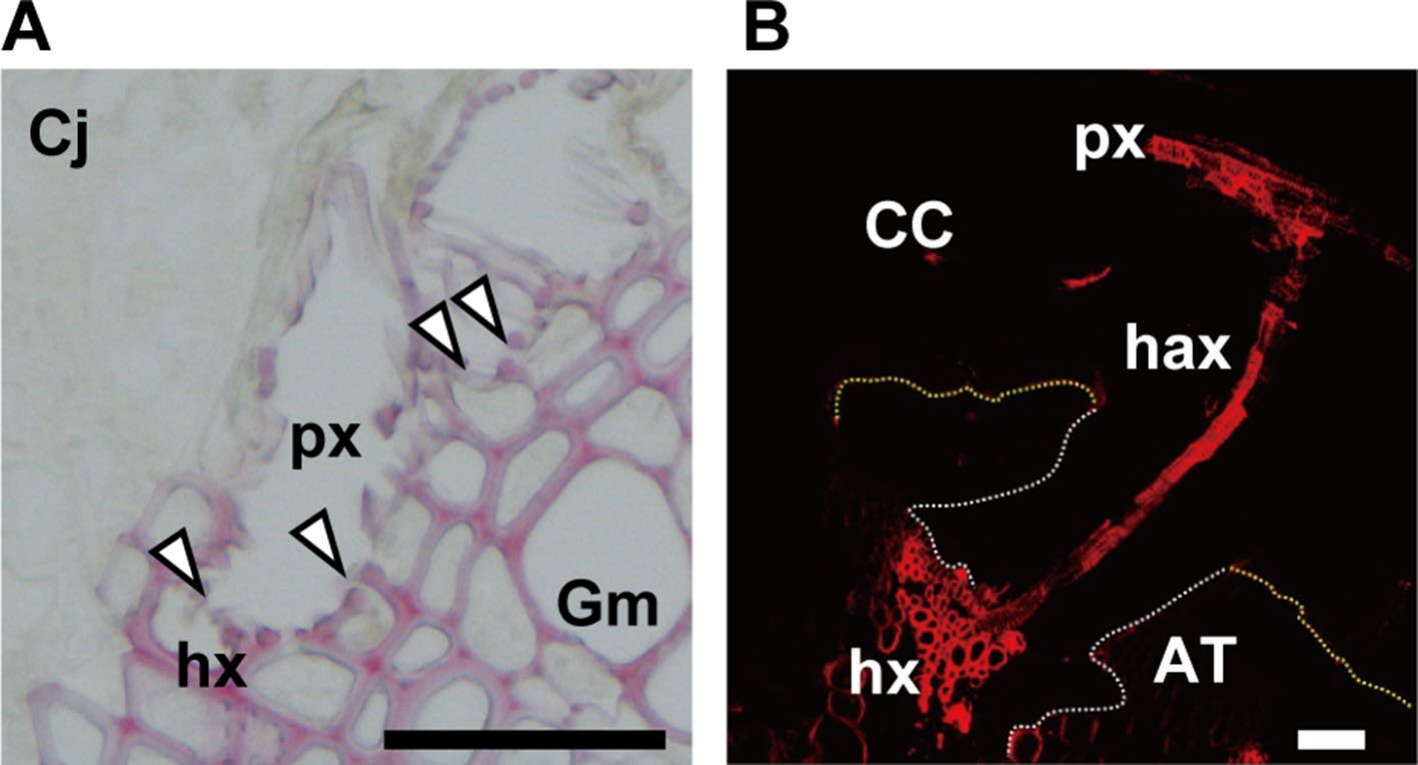
**(A)** Open connection (arrowheads) between xylem vessels of parasite (px) and host (hx) in the parasitic interface of *Cuscuta japonica* (Cj) with *Glycine max* (Gm). Scale bar, 50 μm. A 20-μm-thick paraffin-embedded section was stained with phloroglucinol. **(B)** Transfer of 5-carboxytetramethylrhodamine (TMR) 10-kDa dextran (red) from host xylem vessel to haustorial xylem vessels, and then to *Cuscuta* stem xylem. White dotted line; outline of haustorium, yellow dotted line; outline of attachment boundary between *Cuscuta* and host *Arabidopsis*. CC, *Cuscuta campestris*; AT, *Arabidopsis thaliana*; px, parasite xylem vessel; hax, haustorial xylem vessel; hx, host xylem vessel. Scale bar, 200 μm.

*Cuscuta* becomes a strong sink after the establishment of the haustorial bridge and competes with sink organs of the host itself for assimilates. Searching hypha cells that contact to host xylem vessels invade vessels through the pits in the cell wall (Heide-Jørgensen, 2008). Then, the ends of hypha cell wall become thin and perforated, finally forms an open connection with host xylem vessels (**Figure 4A**). Vaughn (2006) mentioned that the nature of the opening between host xylem and hyphal xylem appears to be dependent on the angle and orientation of hyphae with respect to host xylem. The open connection allows the translocation of xylem-mobile dyes, for example, fluorescently labeled 10-kDa dextran (**Figure 4B**).

The nature of phloem connection has been controversial. Ultrastructural studies demonstrated that phloem continuity is achieved by a contacting searching hyphae which split in finger-formed elongation, and the plasmodesmata and sieve pores are absent between the searching hypha and host sieve tube, suggesting an apoplastic transfer of xylem solutes *via* transfer-type cells (Heide-Jørgensen, 2008). On the other hand, *Cuscuta* has been known as a vector for transmission of virus and phytoplasma (Hosford, 1967; Heintz, 1989), and the transport requires a symplastic connection. Finally, evidence for the presence of symplastic connection was given by the translocation of GFP from sieve tubes of hosts to *Cuscuta* (Haupt et al., 2001). Various phloem-mobile compounds, including sucrose, amino acids, plant hormones, and xenobiotics have been shown to translocate from the host to parasite (Birschwilks et al., 2006). The transport rate does not show any selectivity with respect to the compounds, suggesting that phloem-mobile compounds are transported through an open symplastic connection.

Flow of water from hosts *via* xylem to *Cuscuta* is probably driven by the gradient of water potential between the host and the parasite. In Orobanchaceae, *Orobanche cernua* accumulates a higher level of potassium than the host (Hibberd et al., 1999), and *Striga hermonthica* and *Phelipanche ramosa* accumulates mannitol (Robert et al., 1999). On the other hand, the direction of transport *via* phloem can occur from the parasite to host, and, thus, is bi-directional. The bi-directional nature of phloem transport lays foundations for mutual control between the parasite and the host.

## Interaction With Host

### Host Receptor for Immune Response Against *Cuscuta*

*Cuscuta* spp. have a broad host range, but there are a few plants that are resistant to *Cuscuta* (Kaiser et al., 2015). Interestingly, cultivated tomato species, *Solanum lycopersicum*, is resistant to *Cuscuta reflexa* (Ihl et al., 1988; Albert et al., 2004; Runyon et al., 2010; Kaiser et al., 2015), while a wild relative of tomato, *Solanum pennellii*, is susceptible (Hegenauer et al., 2016; Krause et al., 2018). At the end of the attachment phase, epidermal cells of resistant *S. lycopersicum* die following a hypersensitive-type response, and hypodermal cells are modified to protect intrusion from haustoria (Ihl et al., 1988). Cuscuta factor (CuF), a 2-kDa peptide with *O*-esterified modification, was identified to trigger defense response of the host plant including production of reactive oxygen species and ethylene (Hegenauer et al., 2016). Analysis of introgression lines of *S. lycopersicum* × *S. pennellii* (Eshed and Zamir, 1995) lead to the identification of a gene for tomato receptor of CuF, *CuRe1*, which encodes a leucine-rich repeat receptor like protein (LRR-RLP) (*S. lycopersicum* allele, Solyc08g016270) (Hegenauer et al., 2016). Stable introduction of *S. lycopersicum CuRe1* into susceptible *S. pennellii*, and *N. benthamiana* confers responsiveness to the CuF and increased resistance to *C. reflexa* (Hegenauer et al., 2016). These results suggest that defense response, likely pattern-triggered immunity (PTI) response, of incompatible tomato species could be induced by the perception of the CuF by the receptor CuRe1, although either the molecular identity of CuF or direct binding of CuF to CuRe1 have not been demonstrated yet (Hegenauer et al., 2016). The presence of additional CuRe1-like receptors is also suggested, and the identification of their ligand will pave the way to investigate parasite-host recognition and its relation to plant immunity (Fürst et al., 2016).

### Involvement of Host Factors for Parasitic Organ Development

**Figure 5:**
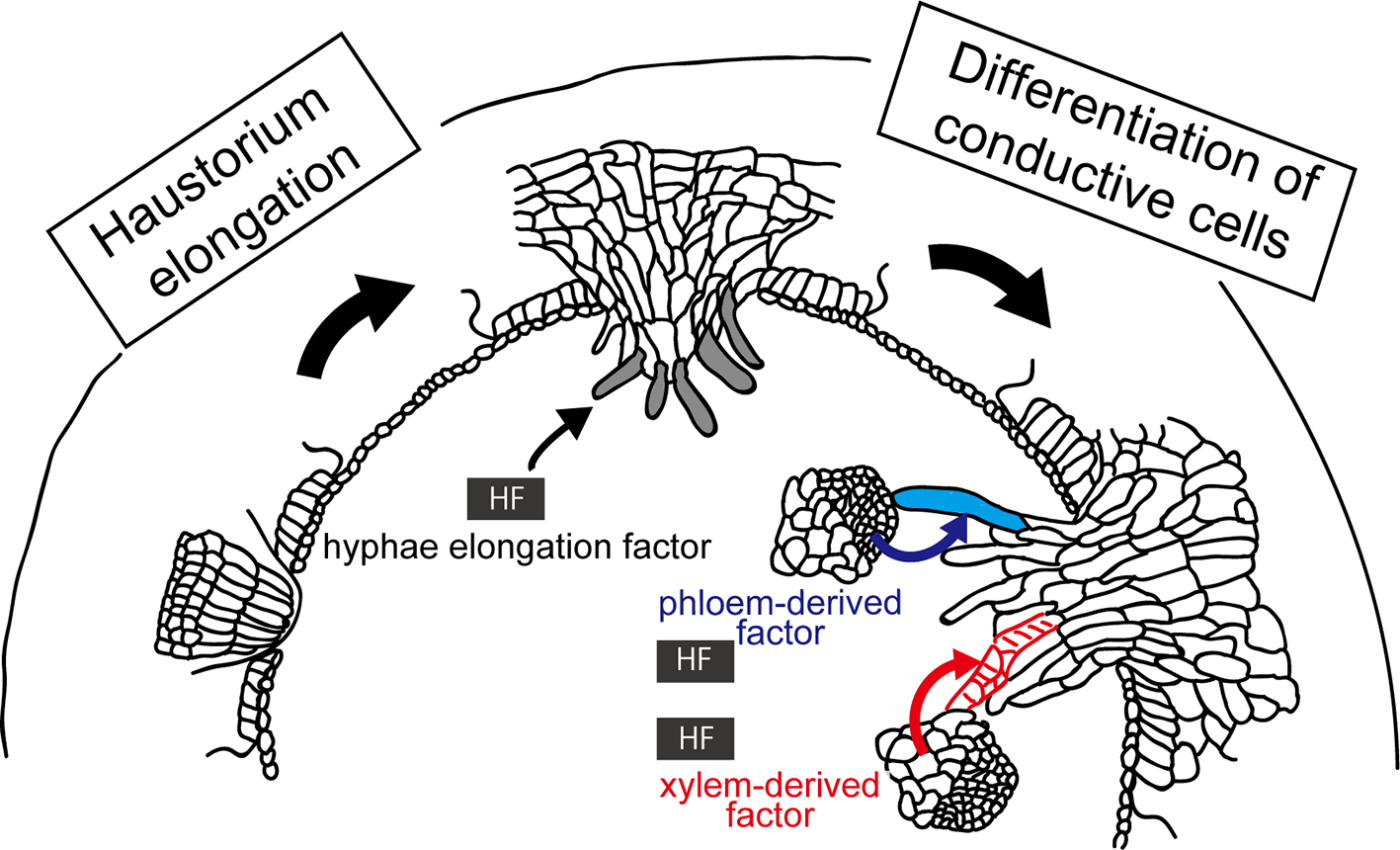
Involvement of host factors (HFs) in the elongation and differentiation of searching hyphae. (Left) HF inducing elongation of searching hyphae (left) has been hypothesized because digitate cells or file cells of *C. campestris* initiated in a host-independent manner do not show further development of searching hyphae without host. (Right) HF has been implied in the differentiation of searching hypha cells into xylem (red lines) and phloem conductive elements (blue), because upon contacting xylem vessels or phloem sieve tubes of the host, the hyphal cells starts to differentiate into respective conductive elements. These HFs have not been identified yet.

Host-derived signal substances, or “host factors,” control the organ development processes of parasites. A well-known example of the host factors are strigolactones, that are exuded from host root, that trigger germination of seeds of Orobancaceae plants (for reviews, see Xie et al., 2010; Lumba et al., 2017). In the case of *Cuscuta*, volatiles emitted from the host is known to mediate host location by *Cuscuta* (Runyon et al., 2006). On the other hand, haustoria can be induced in a host-independent manner (Furuhashi et al., 1995; Tada et al., 1996). Although haustorium initiation can occur host independently, the latter steps, such as elongation of searching hyphae and their differentiation to conductive cells, may require host factors (**Figure 5**).

First, elongation of searching hyphae should be initiated by host factors. The rationale for this is that, although elongation of endophyte primodium of *Cuscuta* initiated by attaching to non-biological substances, such as acryl rod and bamboo stick, develops file cells and digitate cells, they do not show further elongation or development of searching haypha cells (Heide-Jørgensen, 2008; Hong et al., 2011). Host factors involved in this elongation process have not yet been identified.

Second, host factors may be involved in the differentiation of searching hypha cells into xylem and phloem conductive elements (Vaughn, 2006; Krupp et al., 2019). Upon contacting xylem vessels or phloem sieve tubes of the host, the hyphal cells of haustorium starts to differentiate into respective conductive elements, implying that hyphal cells recognize the type of host conductive elements they hit in order to differentiate into the correct elements. Although it is not clear whether this process happens in all cases or not, establishing the right connection between right elements must be essential for the survival of parasitic plants. This raises questions; what the cues of hyphal differentiation are, and whether hyphal cells have multipotency or not. Further study is needed to answer these questions.

We mention that host factors inducing haustorium, or “haustorium inducing factors (HIFs)” are well characterized for Orobancaceae root parasitic plants. Cytokinin (Goyet et al., 2017), 2,6-dimethoxy-1,4-benzoquinone (DMBQ) (Lynn et al., 1981) and lignin-related compounds (Cui et al., 2018) have been shown to have HIF activity. Orobancaceae root parasitic plants may also require host factor(s) for the elongation of intrusive cells because the elongation does not happen when prehaustorium is induced solely by HIFs (Estabrook and Yoder, 1998).

## Parasitic Plants Modulate Organs of the Host?

We so far focused on the organ formation in parasitic plants. On the contrary, modulation of organ morphology by the parasite also occurs in the host plants. Parasitization often causes the swelling of host tissues, which is called “hypertrophy” (Heide-Jørgensen, 2008). In the recent study on the parasitic complex of *Phtheirospermum japonicum* and the host Arabidopsis, thickening of Arabidopsis roots is reported to be induced by the cytokinin produced in *P. japonicum* (Spallek et al., 2017).

In the host plants parasitized by *Cuscuta*, induction of new vascular elements in the host was previously reported (Dawson et al., 1994). However, in *Impatiens balsaminea* parasitized by *Cuscuta pentagona*, little or no new growth of host vascular elements were observed (Vaughn, 2006). In *Glycine max* parasitized by *Cuscuta japonica*, changes in the expression levels were observed for genes responsible for vascular development and cell proliferation, although apparent increase of cell number was not observed in the area adjacent to the invading haustoria (Ikeue et al., 2015). Further study needs to clarify whether invasion of *Cuscuta* affects the morphology of host organ or not.

## Transfer of Long-Distance Signals After Conductive Phase

### RNA Movement

Translocation of mRNAs and small RNAs between *Cuscuta* and the host plant have been shown (Alakonya et al., 2012; LeBlanc et al., 2013) and selectivity of the mobility or uptake of RNA has also been suggested (LeBlanc et al., 2013). A recent study using high-throughput RNA sequencing technology revealed that mRNAs representing more than 8000 genes of the parasite *Cuscuta campestris* and those representing more than 9000 genes of the host Arabidopsis move to the parasitic partner (Kim et al., 2014). Although an unexpectedly large number of RNAs were shown to move from plant to plant, biological relevance and necessity of the movement of mRNAs in the establishment of parasitic relationship are still unclear.

Trans-species movement of small RNAs (sRNA) has been documented for artificially induced short interfering RNA. Trans-silencing of a target gene was employed to demonstrate the role of *SHOOT MERISTEMLESS-LIKE 1* in haustorium development in *C. campestris* (Alakonya et al., 2012). Recently, induction of microRNA (miRNA) was demonstrated in *C. campestris* in the parasitic interface with the host Arabidopsis (Shahid et al., 2018). The miRNAs target transcripts encoding defense-related proteins, such as *AtSEOR1*, *BOTRYTIS-INDUCED KINASE 1* (*AtBIK1*), and members of the *TRANSPORT INHIBITOR RESPONSE 1* (*AtTIR1*)/*AUXIN SIGNALING F-BOX 2* and *3* (*AtAFB2*/*AtAFB3*) family, and accumulation of these transcripts were reduced during parasitization. Although direct evidence for the enhancement of vigor of the parasite has not yet been obtained, the biomass of *C. campestris* on Arabidopsis loss-of-function mutants, *seor1* and *afb3-4*, increased, suggesting that repression of these defense-related genes by miRNAs from the parasite may have biological significance (Shahid et al., 2018).

Trans-species movement of mRNA likely recruit the mechanisms for long-distance movement *via* phloem. Although experimental mRNA mobility can be explained by abundance and half-life of transcripts (Calderwood et al., 2016), presence of sequence motifs such as tRNA-like motifs have been reported selective long-distance movement of mRNA through graft union (Thieme et al., 2015; Zhang et al., 2016). It will be of interest whether the same motifs are functional in the trans-specific movement or not. The mechanisms involved in sRNA transfer needs to be elucidated as well.

### Signals in Response to Herbivory Feeding

Responses to herbivory-feeding in one host plant can transfer to the second host plant connected by the bridging *Cuscuta australis* (Hettenhausen et al., 2017), indicating the feeding signals transfer from host to parasite on the first host, and the other way round on the second host. Feeding by green pea aphid, *Myzus persicae*, induces a local response to *C. australis*, and the signal moves to the soybean host and induces the expression of the herbivory response (Zhuang et al., 2018). These results demonstrate that *Cuscuta* can transmit and receive the systemic signal for herbivory response, although the systemic signal has not been identified yet.

## Conclusion and Future Perspectives

Elucidation of cellular and molecular processes involved in the formation of parasitic organs of *Cuscuta* has unveiled mechanisms hidden in the parasitic interface tissues. *Cuscuta* probably recruits genetic networks shared by other vascular plants, such as the genetic network for protrusive outgrowth of the epidermal cells and for formation of vascular tissues. They use the set of genes in a non-canonical way, though, as seen in the patterning of procambium, xylem, and phloem cells in haustorium. In addition to the formation of parasitic organs, trans-species trafficking of macromolecules, such as RNAs, through parasitic interface suggests a possibility of bi-directional control of biological processes between host and parasite. Understanding of *Cuscuta* will suggest parallels with other multi-organism processes, such as grafting, nematode infection, and formation of insect galls (Melnyk and Meyerowitz, 2015; Viera and Gleason, 2019). Comparative analyses of these processes will reveal the fundamental roles of extracellular and intracellular communication in multi-organism complexes.

## Author Contributions

KS and KA conceived and wrote the manuscript. KS prepared figures. All authors read and approved the final manuscript.

## Funding

This work was supported by Grant-in-Aid for Scientific Research, Grant Numbers 18H03950 and 19H00944 to KA.

## Conflict of Interest

The authors declare that the research was conducted in the absence of any commercial or financial relationships that could be construed as a potential conflict of interest.
